# Timing of antimicrobial use influences the evolution of antimicrobial resistance during disease epidemics

**DOI:** 10.1093/emph/eou027

**Published:** 2014-11-05

**Authors:** Mark M. Tanaka, Benjamin M. Althouse, Carl T. Bergstrom

**Affiliations:** ^1^School of Biotechnology and Biomolecular Sciences, University of New South Wales, Kensington NSW 2052, Australia; ^2^Santa Fe Institute, 1399 Hyde Park Rd., Santa Fe, NM 87501, USA; ^3^Department of Biology, University of Washington, Seattle, WA 98195-1800, USA

**Keywords:** influenza, antiviral, oseltamivir, zanamivir, evolution, drug resistance

## Abstract

**Background:** Although the emergence and spread of antibiotic resistance have been well studied for endemic infections, comparably little is understood for epidemic infections such as influenza. The availability of antimicrobial treatments for epidemic diseases raises the urgent question of how to deploy treatments to achieve maximum benefit despite resistance evolution. Recent simulation studies have shown that the number of cases prevented by antimicrobials can be maximized by delaying the use of treatments during an epidemic. Those studies focus on indirect effects of antimicrobial use: preventing disease among untreated individuals. Here, we identify and examine direct effects of antimicrobial use: the number of successfully treated cases.

**Methodology:** We develop mathematical models to study how the schedule of antiviral use influences the success or failure of subsequent use due to the spread of resistant strains.

**Results:** Direct effects are maximized by postponing drug use, even with unlimited stockpiles of drugs. This occurs because the early use of antimicrobials disproportionately drives emergence and spread of antibiotic resistance, leading to subsequent treatment failure. However, for antimicrobials with low effect on transmission, the relative benefit of delaying antimicrobial deployment is greatly reduced and can only be reaped if the trajectory of the epidemic can be accurately estimated early.

**Conclusions and implications:** Health planners face uncertainties during epidemics, including the possibility of early containment. Hence, despite the optimal deployment time near the epidemic peak, it will often be preferable to initiate widespread antimicrobial use as early as possible, particularly if the drug is ineffective in reducing transmission.

## Abstract (precis)

How can antimicrobial drugs be deployed optimally during infectious disease epidemics? Our mathematical models show it is optimal to delay treatment to maximize successful treatments. In formulating policy, however, this must be balanced against the risk of incorrectly predicting the peak of an epidemic.

## INTRODUCTION

An extensive body of research explores the way in which the schedule of antimicrobial usage is expected to influence the emergence and spread of antibiotic resistance. For example, theoretical models have been developed to address antibiotic resistant strains of *Haemophilus influenzae* and *Streptococcus pneumoniae* in the community [1–3], and methicillin-resistant *Staphylococcus aureus* and vancomycin-resistant enterococci in hospitals [4–13]. For the most part, this body of work deals with endemic disease; only recently have epidemiologists considered the dynamics of resistance evolution in pathogens that undergo epidemic spread. There is a good reason for this historical asymmetry of interest: until recently, we lacked antimicrobials that were effective against common epidemic diseases. The current generation of anti-infleunza therapies—oseltamivir and zanamivir—changes this. These drugs act against seasonal and pandemic influenza, both of which are characterized by epidemic rather than endemic dynamics. Thus, we urgently need to understand how the schedule of antimicrobial use benefits the patient population, and how the evolution of antimicrobial resistance impacts this process.

In doing so, it is important to account for both the direct and the indirect effects of antimicrobial use [14]:

1. The ‘direct effects’ of antimicrobial use accrue from the reduction in mortality and morbidity in treated individuals. Once antimicrobial resistance evolves and spreads, however, further drug use can fail to confer the direct benefit of successful treatment.
2. The ‘indirect effects’ of antimicrobial use manifest as changes in the trajectory of an epidemic. Thus the use of antimicrobials can ultimately alter the total number of cases—treated or otherwise—that occurs over the course of the epidemic.

**Figure 1.**
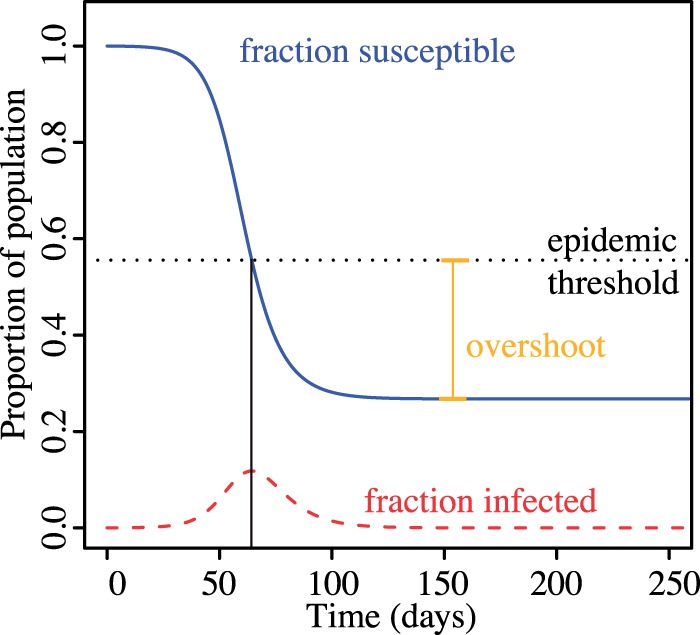
Epidemic trajectory in an SIR model after reference [28]. Overshoot is the number of cases exceeding the minimum cases needed to generate herd immunity

A series of studies has recently addressed the indirect effects of antiviral usage [15–21]. For example, Wu *et al.* [22], Meng *et al.* [23], Handel *et al.* [24], Moghadas *et al.* [25], Althouse *et al.* [26] and Hansen and Day [27] explore optimal schedules of antimicrobial use during an epidemic, but focus on the indirect effect of these drugs, i.e. the resulting changes in the epidemic curves for resistant and sensitive pathogens. (Though Wu *et al.* [22] do acknowledge the importance of having low levels of resistance to maximize antiviral treatment effectiveness, they do not explicitly quantify the direct effects of treatment). Because these studies disregard the direct effects of antimicrobial use on treated individuals, the entire benefit of treatment in those models comes from keeping the effective reproductive number low once herd immunity is generated. That is, in these models, antivirals derive value from reducing the spread of infections late in the epidemic and thereby reduce the amount of ‘overshoot’ [28], beyond the minimum number of cases to establish herd immunity (Fig. 1). (One can infer direct effects from e.g. Wu *et al.* [22] as the difference between the total attack rate and the resistant attack rate while under antiviral treatment, but this is not a focus in that article.)

In this article, we examine how the schedule of antimicrobial use during an epidemic influences direct and indirect effects and then infer how these influences are caused by the timing of resistance evolution. We begin with a model that allows us to track both the direct and indirect effects of antiviral use, and we use it to explore how the timing of drug use affects each type of benefit. We then turn to the specific case of influenza. Based on recent estimates of epidemiological parameters, we argue that direct rather than indirect effects are responsible for most of the benefits of treating seasonal influenza with currently available antivirals. We present an analytical model of resistance evolution during an influenza epidemic, and use this model to show how the timing of antiviral use can be controlled to maximize the direct benefits derived from an antiviral stockpile. For this model, we consider the case in which the antimicrobial does not reduce disease transmission, as has been found for the drug oseltamivir used to treat influenza [29, 30].

## METHODOLOGY

### A model of antiviral resistance evolution

**Figure 2.**
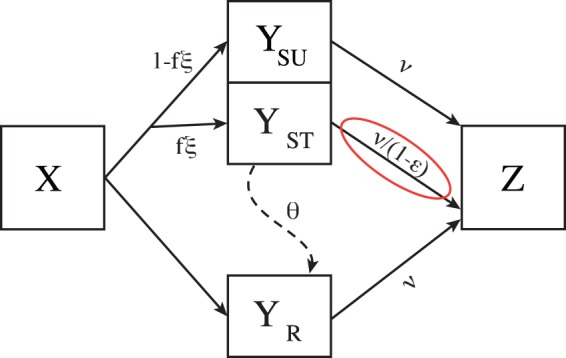
A schematic diagram of the epidemic model. Susceptible individuals (*X*) are infected by drug-sensitive (*Y*_SU_ and *Y*_ST_) and drug-resistant (*Y*_R_) strains by mass action as given in Equation (1). If antimicrobial treatment is ongoing (*ξ* = 1), a fraction *f* of the infected individuals are treated and a fraction 1 − *f* are not; otherwise no individuals are treated. Sensitive treated cases evolve resistance at rate *θ*. Sensitive cases recover at rate *ν* in the absence of drug treatment and at rate ν/(1−ϵ) in the presence of drug treatment; resistant cases recover at rate *ν* irrespective of treatment. All recovered individuals enter the removed class (*Z*). Individuals passing through the red ring increment the tally *A* of successfully treated cases

We model the dynamics of the epidemic using a susceptible-infected-removed (SIR) compartment model, expanded to track sensitive and resistant infection, and treated and untreated patient classes (Fig. 2). In this model, *X* is the fraction of uninfected individuals in the population of size *N*, *Y*_SU_ is the fraction infected with sensitive virus and untreated, *Y*_ST_ is the fraction infected with sensitive virus and treated, *Y*_R_ is infected with resistant virus (treated or not) and *Z* is the fraction of recovered individuals. Resistance evolves in treated individuals infected with drug-sensitive virus at a rate *θ* per case per unit time. We assume that resistance does not evolve in the absence of treatment. Untreated and treated resistant individuals recover from infection spontaneously at a rate *ν*. Treated sensitive cases recover at rate ν/(1−ϵ) so that *ϵ* can be viewed as the reduction in duration of infection due to treatment. Transmission is by mass action, *β* is the transmission parameter, and resistance imposes a transmission cost of *c* on the virus. In addition to reducing the duration of infection via *ϵ*, treatment also reduces the transmissibility of the virus as expressed by the parameter *γ*.

We consider population-level strategies in which the drugs, once initially deployed at time *τ*, are used continuously until they run out or the epidemic is over. During the period of drug treatment, a fraction *f* of the new cases receive the drug. The stockpile of drugs consists of *k* treatment courses. The indirect effects of treatment are given by the number of cases prevented by use of the antimicrobial. The direct effects are given by the cumulative number of successfully treated cases through the end of the epidemic. We include two indicator variables to track drug use: *K* tracks the number of remaining treatment courses starting from *k*, and *A* tracks the number of successfully treated patients starting from zero.

The model, illustrated in Figure 2, is specified by the following system of differential equations.

(1) X˙=−βX(YSU+(1−γ)YST)−β(1−c)XYRY˙SU=(1−fξ)βX(YSU+(1−γ)YST)−νYSUY˙ST=fξβX(YSU+(1−γ)YST)−(ν/(1−ϵ))YST−θYSTY˙R=β(1−c)XYR−νYR+θYSTZ˙=ν(YSU+YR)+(ν/(1−ϵ))YST

where the dot above each state variable indicates a time derivative and where *ξ* = 1 if (t≥τ&K>1) and *ξ* = 0 otherwise. The indicators change according to

(2) $$\begin{array}{l} \dot{K}=-f\xi \beta X(Y_{\text{SU}}+(1-\gamma )Y_{\text{ST}}+(1-c)Y_{R})N\\ \dot{A}=(\nu /(1-\epsilon ))Y_{\text{ST}}N. \end{array}$$

This model structure is similar to that of Lipsitch *et al.* [17] but we do not consider any prophylaxis. Apart from slight differences in parametrization, the other distinguishing features are (i) we include variables that track the number of successfully treated cases (*A*) and the remaining stockpile size (*K*), and (ii) their *de novo* mutation to resistance occurs at transmission while in our model it can occur at any time during infection.

To quantify the indirect effects of treatment, we track the number of cases prevented. Let *ω* be the number of individuals infected by the end of an epidemic (the final epidemic size) in which no treatment is used (i.e. *f* = 0), which is N times the solution of $x=1-e^{-R_{0}x}$ [31]. If $Z_{\infty }$ is the fraction of recovered individuals at the end of the epidemic with treatment, the number of cases prevented by treatment is then $\omega -NZ_{\infty }$. To quantify the direct effects, we track the cumulative number of successfully treated cases through the end of the epidemic, given by *A* as t→∞.

Table 1 provides a summary of the parameters used in this model along with values used in the numerical analysis. Seasonal influenza has a basic reproductive number of around $R_{0}=1.3$ [32] while the pandemic strain of 1918 had an *R*_0_ of 2–3 [33]. We therefore set the basic reproductive number of the disease to $R_{0}=1.8$ corresponding to a strain of influenza that has the potential to cause a pandemic (see also [34]). Influenza typically lasts 6 days [35, 36], giving a recovery rate of γ=0.17 per day. We used a population size *N* of a million to model a medium sized city. The default mutation rate *θ* was set to a low value of 0.01; this is similar to values used by Wu *et al.* [22] which were based on observations that *de novo* resistance occurred in 0.4 and 5.5% of outpatient adults and outpatient children, respectively [37]. The mutation rate and other parameters were given wide ranges to reflect uncertainty in current knowledge (e.g. the efficacy of the drug) or our interest in understanding the effect of varying a parameter (e.g. time of drug deployment, *τ*).

**Table 1.** Parameters of the model

Symbol	Parameter description	Value
$R_{0,SU}$	Basic reproductive number (sensitive, untreated)	1.8
*Ν*	Rate of spontaneous recovery	0.17 per day
*Β*	Transmission parameter	$R_{0}\times \nu$
*c*	Cost of resistance to transmission	0, 0.1
*θ*	Rate of evolution to resistance	0.01 per case per day
*ϵ*	Reduction in infection duration due to treatment	0, 0.5
*γ*	Reduction in disease transmission due to treatment	0, 0.5
*τ*	Time at which drugs are deployed	60 days
*f*	Fraction of new cases receiving drug	0.5
*N*	Population size	10^6^
*k*	Drug stockpile size	*N* or 0.1 *N*

## RESULTS

### Numerical analysis

**Figure 3.**
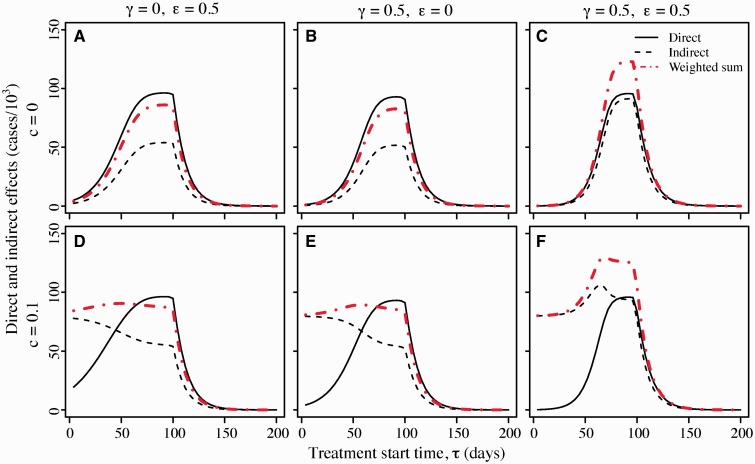
Effects of antimicrobial therapy as a function of the time at which treatment is deployed. Direct effects (solid line) are measured in thousands of cases treated successfully, i.e. by treatment of individuals infected with drug-sensitive strains. Indirect effects (dashed line) reflect the change in the epidemic trajectory due to antimicrobial use: the indirect effects of treatment are quantified by the decrease in the number of cases in thousands when treatment is used relative to the number that would have occurred in the absence of treatment. The weighted sum of effects (indirect effects + 0.33*direct effects) is shown with red dot-dashed lines. In the top panels **(A–C)** the cost of resistance *c* is zero; in the bottom panels **(D–F)** cost *c* = 0.1. A and D: treatment reduces duration of infection (ϵ=0.5) but not transmissibility (*γ* = 0). B and E: treatment reduces transmissibility (γ=0.5) but not duration (*ϵ* = 0). C and F: treatment reduces duration (ϵ=0.5) and transmissibility (γ=0.5). Unless indicated otherwise, the other parameters are as given in Table 1 with stockpile size k=0.1N

Figure 3 shows the direct and indirect effects of treatment as a function of the time at which antimicrobial treatment is initiated. The six panels illustrate six different parameter sets. In all six panels, there is a limited stockpile with doses enough to cover only one 10th of the total population. In the top row panels, there is no cost of resistance, whereas in the bottom row resistant strains suffer a 10% transmission cost. In the left column, drug use reduces the duration of infectiousness, in the center column drug use reduces transmissibility, and in the right column drug use reduces both duration and transmissibility.

Indirect and direct effects are not equivalent. Indirect effects represent cases entirely avoided, whereas direct effects represent cases that occur but are successfully treated. A health planner faced with a pandemic should aim to maximize neither the direct effects by themselves, nor the indirect effects by themselves. Rather, a planner would typically aim to maximize some weighted sum of the direct and indirect effects, where the weighting *α* of direct effects reflects the value of a case successfully treated relative to a case avoided entirely. The panels in Figure 3 therefore show a weighted sum of direct and indirect effects as well, where the weight is chosen as α=0.33 to reflect treatment’s reduction in risk of lower respiratory complications [38]. A number of general results emerge.

**Figure 4.**
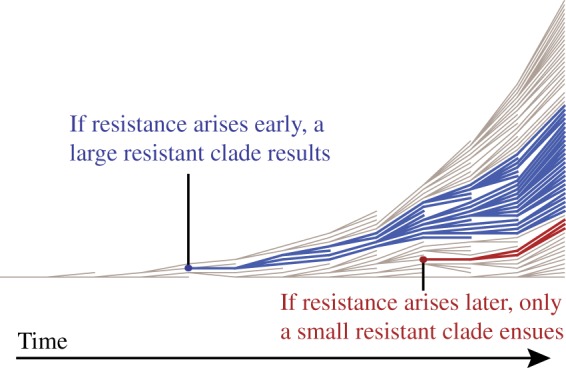
The timing at which the first resistant clade arises has a strong impact on the subsequent prevalence of resistant strains in the population. If the first resistant strain arises early in the epidemic, a large resistance clade (blue) is created. If the first resistant strain arises after several generations of transmission, the resulting resistant clade (red) is far smaller

First, in all cases the direct effects of antimicrobials are maximized by postponing the onset of antimicrobial usage until well into the epidemic. These results derive from a simple observation about resistance dynamics in an epidemic setting: the timing of the appearance of initial resistant clades will have a major effect on the subsequent prevalence of resistant strains in the population. Figure 4 illustrates this principle. When resistance evolves early in a growing population, a larger clade results than when resistance emerges late in the epidemic. Deploying drugs right from the onset of the epidemic risks early evolution of resistance and thus takes the chance that a large fraction of the epidemic cases will be resistant. Assuming that the initial cases are drug sensitive, postponing drug use for a few weeks gives sensitive clones a sufficient head start that the large majority of cases in the epidemic will be drug sensitive. (Note the analogy between evolution of resistance in this model and mutation in the Luria-Delbruck process [39]. Under the Luria-Delbruck process, the number of mutants arising in an exponentially growing bacterial culture has a skewed distribution with a high variance. This high variation is precisely due to the unpredictability of the timing of mutations during exponential growth, where early mutation events lead to large clades and later mutation events lead to small clades.) We further note that the reduced direct effect from early deployment is also partially due to the fact that early treatment limits the number of sensitive cases when the drug affects transmission. If treatment is started too late, the epidemic will conclude before the stockpile has been exhausted and the unused courses will be wasted. As a result, there is an intermediate optimum time to initiate treatment.

Second, when there is no cost of resistance, the indirect effects are also maximized by postponing the onset of antimicrobial use. Assuming that the epidemic cannot be contained, indirect effects result from minimizing the degree of overshoot, i.e. minimizing the number of cases beyond the number that would be required to reach the epidemic threshold (Fig. 1). If drugs are deployed too early, the stockpile will be exhausted before reaching the epidemic peak and large resistant clades will render treatment less effective at reducing the effective reproductive number; if drugs are deployed too late, treatment courses will go unused. Thus again we see an intermediate optimum time to start treatment near the epidemic peak, as noted by Hansen and Day [27], Wu *et al.* [22] and Althouse *et al.* [26].

Third, when resistance imposes fitness costs, indirect effects can be larger when treatment is initiated early. This effect arises because the resistant strains now have relatively ‘low’ fitness once the drugs run out, so that starting early and producing large resistant clades reduces the overall size of the epidemic. Looking at the weighted sum of direct and indirect effects, the optimal times again shift to later in the epidemic but the cost of waiting is often minimal.

Fourth, the effects of antimicrobial use are similar whether they come about through a reduction of duration or a reduction of transmissibility.

**Figure 5.**
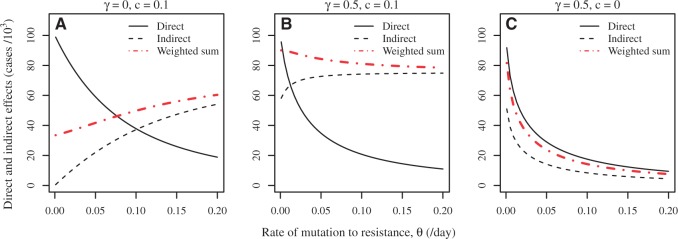
The effect of rate of mutation to resistance (*θ*) on direct and indirect effects. **A:** the drug does not reduce disease transmission (*γ* = 0) and resistance comes with cost *c* = 0.1; **B:** the drug does reduce disease transmission (γ=0.5) and resistance comes with cost *c* = 0.1; **C:** the drug reduces transmission (γ=0.5) but resistance is cost-free (*c* = 0). Treatment start time *τ* is set to 60 days; the efficacy of the drug to reduce infection duration is *ϵ* = 0; stockpile size is k=0.1N; the remaining parameters except *θ* are as given in Table 1

We have also investigated the effect of the *de novo* mutation rate *θ* on direct and indirect effects and their weighted sum (Fig. 5) Low mutation rates lead to few resistant clades and therefore more successfully treated cases—high direct effects—while high rates lead to large resistant clades and low direct effects. However, the resistance mutation rate has the opposite effect on indirect effects when there is a transmission cost of resistance. This is because resistant strains are less fit than sensitive strains and therefore the final epidemic size is lower with more resistant viruses. When there is no resistance cost but the drug reduces transmission, there is a greater indirect effect under low mutation rates because the drug is effective in preventing cases when there are more sensitive cases.

### Application to influenza

In this section, we apply the model to influenza A virus and treatment with the antiviral oseltamivir (Tamiflu). Although oseltamivir can shorten the duration of illness and reduce the severity of symptoms [40–42], Yang *et al.* [29] have argued that unlike osteltamivir prophylaxis, oseltamivir treatment of infected cases has little or no ability to prevent transmission (see also [30, 43, 44]). This finding makes sense because most transmission occurs before treatment has substantive effect on viral titre [18].

**Figure 6.**
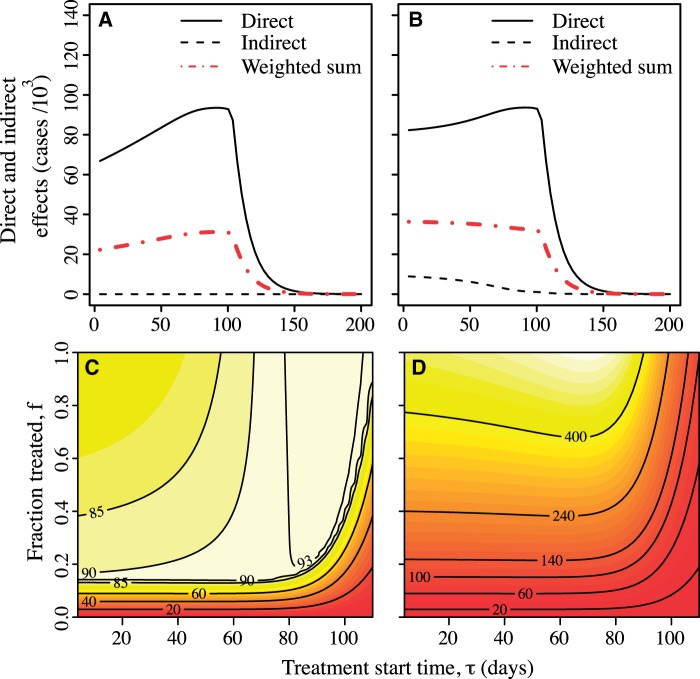
Effects under zero effect of treatment on transmission rate (*γ* = 0) and recovery time (*ϵ* = 0). **A, B**: direct and indirect effects and weighted sum as a function of the starting time *τ* with no cost *c* of resistance (panel A) and a small cost, *c* = 0.1 (panel B). Here, the fraction treated is *f* = 0.5. **C, D**: direct effects of drugs as a function of both treatment start time *τ* and proportion of cases treated *f*, with limited stockpile (k/N=0.1, panel C) and unlimited stockpile (k/N=1, panel D) of drugs. Here, the cost of resistance is set to zero. The other parameters are as given in Table 1: recovery rate ν=0.17, mutation rate θ=0.01, population size $N=10^{6}$, basic reproductive ratio for susceptible untreated cases $R_{0,SU}=1.8$; *β* is computed from $R_{0,SU}$ and *ν*. The effects are given in units of thousands of cases

If a drug has no effect on transmission, the indirect effects of treatment are zero but the direct effects may be substantial. Figure 6 shows direct effects in the case where the drug is not effective in reducing either transmission or duration of infection (*γ* = 0, *ϵ* = 0). Panel A shows that when drug efficacy in reducing transmission is zero, the indirect effects are zero, but direct effects remain high. Again the optimal time to start treatment according to the direct criterion is near the peak of the epidemic. (Panel B) shows the effects of treatment when there is a small cost of resistance. Figure 6 explores the direct effects of treatment for limited stockpiles (Panel C) and unlimited stockpiles (Panel D) when varying both starting time *τ* and the proportion of cases treated, *f*. Whether or not doses are limited, the optimal start time is still near the peak of the epidemic. Starting earlier results in a mild decrease in direct effects but this decrease is much smaller than the case in which antimicrobials can reduce transmission (Fig. 3) because in that case the size of the sensitive outbreak is reduced by early deployment.

In the Appendix 1, we present an analytical model to show how and why these effects arise.

## DISCUSSION

The theory of how antimicrobials should be used in a population differs in character between epidemic and endemic disease. In epidemics, case numbers rise approximately exponentially at first before declining to low numbers. There is a need to reconsider the optimization of control strategies under such conditions. By considering the indirect effects of antimicrobial use, recent work has found that the optimal time to commence treatment in a population is well into the course of an epidemic [21, 22, 26, 27]. We have examined a different effect of drugs: the direct effects of successful treatment. Our analysis shows that delaying the deployment of drug treatments in a population increases the number of successfully treated cases even if there is an unlimited stockpile of a drug. The reason for the advantage of delay is that it prevents large clades of resistant virus from arising early in the epidemic. The size of a resistant clade depends on the number of cases in the population at the time a resistant mutant appears. It is also strongly influenced by the rate at which resistance arises *de novo* by mutation. Delaying treatment until near the epidemic peak keeps the relative frequency of drug resistant infections low.

How much does it matter whether treatment starts near the peak of the epidemic? Where antimicrobials have little efficacy in reducing transmission, which may apply to influenza A [29, 44], the direct effects are not much lower than if drugs start to be deployed near the beginning of the epidemic compared with starting deployment near the peak (Fig. 6). Because sensitive strains have equal fitness to resistant strains, as the epidemic initially grows exponentially there is a large and growing pool of (sensitive) cases that can successfully be treated. Although this sensitive pool would be larger if drugs are deployed later in the epidemic, on average it would not be much larger as long as the mutation rate is low.

Other considerations also argue against excessively delaying the deployment of antimicrobials. Early on in the course of an epidemic there is considerable uncertainty about its future trajectory. Misestimating the course of the disease and waiting too long to initiate drug use carries the risk of failing to use the full stockpile before the epidemic is over. This is arguably worse than running out of drugs before the epidemic is finished [20]. Early in an epidemic, there may be other reasons for immediate and aggressive use of available drugs. Most importantly, there may be a non-zero probability of successful containment and eradication of the outbreak if the drug reduces transmission sufficiently or if other effective measures can be applied [34, 45, 46]. Withholding available antiviral treatment from individual patients who could potentially benefit from their use also poses an ethical problem, pitting the rights of the individual against the good of the collective. On the balance, we expect that in most situations immediate use of a stockpile is likely to be the best approach. If stockpiles of two drugs are available, the approach proposed by Wu *et al.* [22] may be particularly effective: use the smaller stockpile first as a way to delay the use of the larger stockpile and thus delay the evolution of resistance to this latter drug. In any event we will rarely if ever detect the very first cases of an epidemic in real time; by the time a problem is identified the epidemic may have progressed far enough that immediate use of the stockpile will be optimal.

## CONCLUSIONS AND IMPLICATIONS

This study distinguishes between the direct and indirect effects of deploying antimicrobial drugs. The indirect effects of lowering the final epidemic size—that is, averting cases—are large whenever the antimicrobials have substantial efficacy in reducing transmission or duration of disease. This benefit is often taken to be an important objective of disease control strategies [e.g. 27], but planners will also want to consider the direct effects of antimicrobial use on infected patients. In particular, when antivirals have little effect on transmission, there is little indirect effect but the direct effects of treating cases successfully can be substantial. These direct effects accrue as long as resistant clades are small and available treatments are used before the epidemic ends. The direct effects count the treatment of sensitive cases regardless of whether drugs change the epidemic trajectory. In principle, a particular usage policy might result in more successful instances of treatment because it has the highly undesirable consequence of creating a larger epidemic and therefore offering more patients to treat. Thus maximizing direct effects should not be used alone as an objective in disease control planning. Instead, planners will typically want to make decisions based on a composite of both direct and indirect effects.

## Figures and Tables

**Figure 7.**
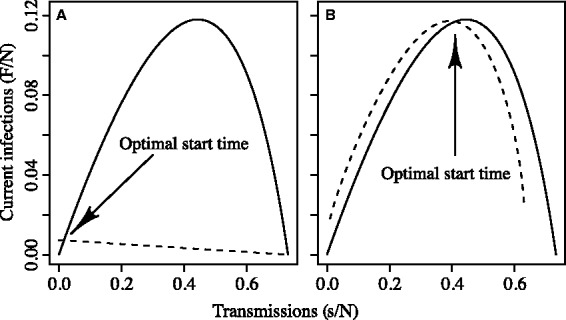
Solutions for the optimal starting time of treatments $\hat{\sigma }$ when the supply of the drug is **(A)** unlimited and **(B)** limited. In each case the epidemic trajectory F(σ) (solid curve) is scaled by the reciprocal of the population size, 1/*N*. Treatment start time *σ* is also scaled by 1/N. **A**: unlimited stockpile. The dashed line is (ω−σ)μ which intersects with the epidemic trajectory F(σ) (solid curve) at the optimum, $\hat{\sigma }$. **B:** limited stockpile: the dashed curve is the harmonic mean number of cases from the start of treatment in the population until the drug runs out. This curve intersects the epidemic trajectory *F*(*s*) (solid curve) at the optimum, $\hat{\sigma }$. This example uses a standard SIR model with no birth or death. The parameters here are as given in Table 1: $R_{0}=1.8,\nu =0.17,\mu =0.01,k/N=0.1$

## APPENDIX 1

### ANALYSIS OF A SIMPLIFIED MODEL

Here, we analyse a simplified version of the model for the case in which the drug does not affect transmission though it may reduce symptoms. In the Results, we discuss how these conditions apply to treatment of influenza A virus with oseltamivir.

We begin with a general function describing the trajectory of the epidemic, and then investigate how the timing of treatment influences the evolution and spread of resistance and the consequent instances of treatment failure. In the analytical model, as in Figure 6, treatment does not reduce transmission; neither the timing of resistance evolution nor the schedule of antiviral therapy exerts an influence on the net trajectory of the epidemic. The value of drug use lies entirely in reducing the morbidity suffered by the treated individual. There is no transmission cost of resistance and no selective differential operating between resistant and sensitive strains.

We define the epidemic trajectory *F*(*s*) as the current number of infectious cases in a population after *s* transmission events have taken place. Note that by parameterizing this curve in terms of cumulative transmission events *s* rather than calendar time *t*, we use a variable-speed clock that ticks every time a new case occurs. This approach considerably simplifies the analysis. We further assume that once treatment is initiated, all infected cases are treated (*f* = 1) until the drug supply is exhausted.

#### The epidemic trajectory

To provide an example of the epidemic trajectory function *F*(*s*) we use an SIR model without births or deaths [31]. The parameters of this model are the total population size *N*, the transmission coefficient *β* and the recovery rate *ν*. Let *X*, *Y*, *Z* track the proportion of susceptible, infectious and recovered individuals. We do not differentiate between resistant and sensitive strains here. The differential equations for this process are

dXdt=−βXYdYdt=βXY−νY

The fraction of recovered individuals, *Z* is 1−(X+Y). The basic reproductive number is $R_{0}=\beta /\nu$.

Now define s=N(Y+Z)=N(1−X), which tracks time through transmissions. This transmission-counting variable “ticks” at each transmission event. The above system can be rewritten as

dsdt=β(N−s)YdYdt=β(N−s)Y/N−νY

and so

$$\frac{dY}{ds}=\frac{dY/dt}{ds/dt}=\frac{1}{N}-\frac{\nu }{\beta (N-s)}=\frac{1}{N}-\frac{1}{R_{0}(N-s)}$$

for Y≠0. The solution of this differential equation with initial condition Y(0)=1/N is $\left(1+s)/N+(1/R_{0})\log(1-s/N\right)$. Thus, for this model the epidemic trajectory is

(3) $$F(s)=NY(s)=1+s+\frac{N}{R_{0}}\log\left(\frac{N-s}{N}\right).$$

#### Unlimited doses

First, consider the case in which health planners have access to an unlimited stockpile of the antiviral. We consider the case in which drug resistance and sensitivity are neutral: there is no advantage to sensitive virus in the absence of treatment (*c* = 0) or to resistant virus in the presence of treatment (γ=ϵ=0). Let *ω* be the final size of the epidemic, *μ* be the probability of mutation to resistance per transmission, and *σ* be the case number at which treatment is initiated in the population. A mutant appears at case number *s* with probability *μ* and at frequency of 1/F(s) which will remain unchanged for the remainder of the epidemic on average because it is selectively neutral. At case *s* there are ω−s remaining cases in the epidemic and therefore the mutant at *s* will have (ω−s)/F(s) descendants. The direct effects of antiviral use, denoted by Ψ, is the number of successfully treated cases which is approximately

(4) $$\Psi (\sigma )\approx (\omega -\sigma )-\int_{\sigma }^{\omega }\mu \frac{\left(\omega -s\right)}{F(s)}\text{d}s.$$

Here the (ω−σ) term represents the total number of treated cases, successful or unsuccessful, and the integral represents the number of treatment failures due to resistance. The approximation assumes that mutation from sensitivity to resistance is rare and does not directly alter the number of drug sensitive cases.

We aim to select a starting point *σ* to maximize the number of successful treatments. If an interior maximum exists, a necessary condition is that dΨ/dσ=0. To evaluate this derivative of Ψ, we apply the Leibniz rule for differentiation under the integral sign:

$$\frac{\text{d}\Psi }{\text{d}\sigma }=\frac{(\omega -\sigma )\mu }{F(\sigma )}-1.$$

Thus the extremum $\hat{\tau }$, if it exists, occurs at $\sigma =\hat{\sigma }$ where

(5) $$F(\hat{\sigma })=(\omega -\hat{\sigma })\mu .$$

This extremum is a maximum; the second derivative is

$$\frac{{\text{d}}^{2}\Psi }{\text{d}\sigma^{2}}=\frac{-\mu F(\sigma )-\frac{\text{d}F(\sigma )}{\text{d}\sigma }(\omega -\sigma )\mu }{F{\left(\sigma \right)}^{2}}.$$

The quantities *μ*, F(σ), and (ω−σ) are all positive; dF(σ)/dσ is also positive at $\sigma =\hat{\sigma }$, in the case of an epidemic with a single peak, as illustrated below. Therefore for any $\hat{\sigma }$, the second derivative ${\text{d}}^{2}\Psi /\text{d}\sigma^{2}<0$ and Ψ is maximized at $\hat{\sigma }$.

The epidemic trajectory *F*(*s*) can take any functional form, but we illustrate in Figure 7A how the analysis is applied to the standard SIR model [31] using Equation (3). The straight line in the right hand side of Equation (5) has a positive intercept ωμ and crosses the rising epidemic curve F(σ). Because many cases occur rapidly near the peak of the epidemic, this intercept is still relatively late in the epidemic in terms of calendar time units. Our model reveals that even with an unlimited supply of the drug, it is not optimal to start treatment in the population at the beginning of the epidemic. Starting treatment too early creates large clades of resistant viruses; as a result many treatment failures will occur.

#### Limited doses

Second, we consider the case in which the number of antiviral treatment courses is limited. Here, we derive an expression for the number of successful treatments Ψ with a minor modification of Equation (4). If *k* courses of treatment are available and all are used before the end of epidemic, the number of successful treatments is given by

(6) $$\Psi (\sigma )=k-\int_{\sigma }^{\sigma +k}\mu \frac{\left(\sigma +k-s\right)}{F(s)}\text{d}s.$$

If doses still remain after the epidemic has ended, the number of successful treatments is again given by Equation (4). Otherwise, to find the optimal case number on which to initiate treatment, we consider the derivative with respect to *σ*, again applying Leibniz’s rule so that

$$\frac{\text{d}\Psi }{\text{d}\sigma }=\mu \left(\frac{k}{F(\sigma )}-\int_{\sigma }^{\sigma +k}\frac{1}{F(s)}\text{d}s\right).$$

This derivative is zero when

$$F(\hat{\sigma })=\frac{k}{\int_{\hat{\sigma }}^{\hat{\sigma }+k}[1/F(s)]ds}.$$

This relation holds when the number of infectious cases at the start of treatment in the population is equal to the harmonic mean of the number of cases until the drug runs out.

With a single epidemic peak, this implies that $\hat{\sigma }$ is just left of the peak: *F*(*t*) must rise from that point, then drop below $F(\hat{\sigma })$. Therefore, $F(\hat{\sigma })>F(\hat{\sigma }+k)$. The second derivative is

$$\frac{{\text{d}}^{2}\Psi }{\text{d}\sigma^{2}}=\mu \left(\frac{-k}{F{\left(\sigma \right)}^{2}}-\left(\frac{1}{F(\sigma +k)}-\frac{1}{F(\sigma )}\right)\right)$$

which is negative at $\sigma =\hat{\sigma }$ because μ,k,F(σ) are all positive and because $F(\hat{\sigma })>F(\hat{\sigma }+k)$. Figure 7B illustrates the position of the optimal start time $\hat{\sigma }$ near the peak of the epidemic. If there were multiple waves in the epidemic curve *F*(*s*), both conditions on the derivatives would only hold for the peaks.

The optimal placement of *σ* is near the peak because that is when the frequency of resistant lineages will be the lowest when they arise (i.e. 1/F(s)). By this heuristic argument, it is clear that for a single epidemic wave it is optimal to place all treatment courses into a single contiguous block near the peak rather than say (i) treating only a fraction of cases once the drug starts to be used or (ii) treating all people in discontinuous blocks of time (Hansen and Day [27] show this result for indirect effects using different models and methods).

If the rate of mutation to resistance is high, the probability of early emergence of resistance is high along with the risk of large resistant viral clades. Although the mutation rate does not affect the optimal time to start treating cases, the higher the mutation, the stronger the need to start treatment near the epidemic peak to minimize the impact of treatment failure. This can be understood mathematically through the second derivative of the direct effects expression Ψ(σ) which is proportional to the mutation rate.
